# The neutrophil-to-lymphocyte and platelet-to-lymphocyte ratios predict efficacy of platinum-based chemotherapy in patients with metastatic triple negative breast cancer

**DOI:** 10.1038/s41598-018-27075-z

**Published:** 2018-06-07

**Authors:** Claudio Vernieri, Alessia Mennitto, Michele Prisciandaro, Veronica Huber, Monica Milano, Lucia Rinaldi, Maria Silvia Cona, Claudia Maggi, Benvenuto Ferrari, Siranoush Manoukian, Gabriella Mariani, Giulia Bianchi, Giuseppe Capri, Licia Rivoltini, Filippo de Braud

**Affiliations:** 1 Medical Oncology Unit, Fondazione IRCCS Istituto Nazionale dei Tumori, Via Venezian 1, 20133 Milan, Italy; 2 Fondazione Istituto FIRC di Oncologia Molecolare (IFOM), Via Adamello 16, Milan, Italy; 3 Immunotherapy of Cancer Unit, Fondazione IRCCS Istituto Nazionale dei Tumori, Via Venezian 1, 20133 Milan, Italy; 4 Medical Genetics Unit, Fondazione IRCCS Istituto Nazionale dei Tumori, Via Venezian 1, 20133 Milan, Italy; 5 Universita’ degli Studi di Milano, Milan, Italy

## Abstract

Platinum salts are active against metastatic triple negative breast cancer (mTNBC), and biomarkers to predict their effectiveness are urgently needed. In recent years, the neutrophil-to-lymphocyte ratio (NLR) and the platelet-to-lymphocyte ratio (PLR) have emerged as prognostic biomarkers in many malignancies, but their predictive role in platinum-treated mTNBC patients remains unexplored. We performed a retrospective, single centre study to evaluate the association between baseline NLR or PLR and progression free survival (PFS) of mTNBC patients treated with platinum-based chemotherapy. As a control population, we analysed data from patients with hormone receptor-positive HER2-negative (HR+ HER2−) metastatic breast cancer. Among 57 mTNBC patients treated with the carboplatin-paclitaxel or carboplatin-gemcitabine combination, high NLR and PLR were associated with significantly lower PFS at both univariate and multivariable analysis. Conversely, we did not find a significant association between NLR or PLR and the PFS of 148 patients in the control population. Our findings suggest that the NLR and PLR are predictive of benefit from platinum-containing chemotherapy specifically in mTNBC patients. If validated in larger prospective studies, these easy-to-measure parameters could be combined with emerging predictive biomarkers, such as *BRCA 1/2* mutations, to improve the selection of mTNBC patients more likely to benefit from platinum-based chemotherapy.

## Introduction

Breast cancer (BC) is the most common malignancy among women and also one of the leading causes of cancer-related death^1^. Metastatic triple negative breast cancer (mTNBC) accounts for 10–20% of metastatic BC (mBC) cases, and is characterized by an aggressive course, the lack of therapeutic targets and high lethality^2,3^. Even in recently published prospective trials, median progression free survival (PFS) and overall survival (mOS) did not exceed 6 months and 12–18 months, respectively^4,5^.

Cytotoxic chemotherapy (ChT) remains the mainstay of treatment for mTNBC, with taxane and platinum salts being among the most effective compounds when used alone, or in combination with bevacizumab or other chemotherapeutical agents^4–9^. In a recent randomized study, carboplatin demonstrated superior activity compared to docetaxel in mTNBC patients bearing germline mutations in *BRCA1* or *BRCA2* genes, thus suggesting that platinum compounds should be preferred treatment options in this patient population^6^. While such results await confirmation in larger studies, new predictive biomarkers are needed that are low-cost, routinely assessable with standardized techniques, well reproducible across different laboratories, and capable of predicting benefit from platinum-based ChT.

Many evidences highlight the importance of immune system activation in TNBC control: first, tumor-infiltrating lymphocytes (TILs) and other immune markers correlate with higher rates of pathological complete response (pCR), as well as with better patient disease-free survival (DFS) and OS, after neoadjuvant ChT^10–13^; second, the programmed death 1 (PD-1) inhibitor pembrolizumab and the PD-1 ligand (PD-L1) inhibitor atezolizumab are active in subgroups of heavily pre-treated TNBC patients^14–16^; lastly, combining nab-paclitaxel with atezolizumab has shown promising clinical activity in TNBC^17,18^, and taxanes-atezolizumab combinations are being evaluated in larger prospective trials [NCT02425891; NCT03125902].

Recent studies have revealed the prognostic role of parameters that reflect systemic inflammation or the status of antitumor immunity, such as the neutrophil-to-lymphocyte ratio (NLR) and the platelet-to-lymphocyte ratio (PLR), in different solid malignancies, including colorectal, renal and lung carcinomas^19,20^. High NLR has also been associated with an increased risk of death in heterogeneous BC patient populations^21^, including patients with limited-stage TNBC^22,23^. However, none of these studies specifically assessed the role of NLR in mTNBC and, even in those studies including different proportions of patients with mBC (4.2–13.9%), subgroup analyses in specific tumor biology subgroups, or based on the type of ChT, were not reported^24–28^.

In this work, we assessed for the first time the potential role of NLR and PLR as biomarkers predictive of PFS in mTNBC patients treated with platinum-based ChT. As a control population, we analysed data from patients with hormone receptor-positive HER2-negative (HR+ HER2−) mBC treated with the same platinum-containing regimens. To discriminate between a predictive and a purely prognostic role of these parameters, we also evaluated patient OS.

## Results

### Patient characteristics

Between July 2007 and July 2017, 62 mTNBC patients were treated at our Institution with platinum-based ChT combinations. Among these patients, 57 fulfilled the inclusion criteria and were evaluable for the biomarkers of interest. At the moment of data lock and analysis, 53 (93%) patients had progressed and 48 (84%) had died.

Characteristics of evaluated patients are described in Table 1. Median age among TNBC patients was 56 years (range 33.7–78.9). Most patients (84%) received carboplatin-paclitaxel ChT, while the remaining ones (16%) were treated with the carboplatin-gemcitabine combination. All mTNBC patients received platinum-containing ChT as their first- (88%) or second-line (12%) treatment.

**Table 1:** Characteristics of patients with mTNBC and the HR+ mBC control population.

	TNBC	ER/PgR-positive HER2-negative
N. pts	57	148
Median age (years, range)	56 (33.7–78.9)	58 (29.8–79.3)
Previous taxane exposure		
Yes	43 (75.4%)	102 (68.9%)
No	14 (24.6%)	46 (31.1%)
N. chemotherapy line		
1^st^–2^nd^	57 (100%)	104 (70.3%)
>2^nd^	0 (0%)	44 (29.7%)
N. disease sites:		
1–2 sites	36 (63.2%)	88 (59.5%)
>2 sites	21 (36.8%)	60 (40.5%)
Presence of visceral disease	37 (65%)	99 (66.9%)
Type of treatment		
paclitaxel	48 (84.2%)	112 (75.7%)
gemcitabine	9 (15.8%)	36 (24.3%)
Maintenance therapy	14 (24.6%)	65 (43.9%)

In the control population of HR+ HER2− patients, median age was 58 years (range 29.8–79.3); most patients (75.7%) were treated with carboplatin-paclitaxel and the remaining ones (24.3%) received carboplatin-gemcitabine. Of these patients, 70.3% received platinum-containing ChT as their first- or second-line therapy.

### Impact of NLR and PLR on PFS

Median PFS in the TNBC population was 204 days. Higher neutrophils (4000/µl) were associated with non-significantly lower PFS (p = 0.081), while platelets above 300000/µl or lymphocytes below 1500/µl correlated with significantly shorter PFS (p = 0.041 and p = 0.005, respectively) (Fig. S1).

We then investigated the potential predictive role of NLR and PLR. Based on previous studies, we chose a NLR threshold of 2.5 and a PLR threshold of 200^21,29^. Both NLR and PLR were inversely associated with patient age (p < 0.001 and p = 0.048, respectively), while they did not correlate with previous taxane exposure (p = 0.076 and p = 0.68, respectively), presence of visceral disease (p = 0.9 and p = 0.6, respectively), type of ChT received (p = 0.48 and p = 0.7, respectively) and number of metastatic sites (p = 0.22 and p = 0.39, respectively) (Table 2). Finally, high NLR correlated with lower probability of receiving maintenance ChT (p = 0.017), while PLR did not (p = 0.097).

**Table 2:** Characteristics of mTNBC patients by NLR and PLR.

	Total (n = 57)	NLR <2.5 (n = 25)	NLR ≥2.5 (n = 32)	p value	PLR <200 (n = 34)	PLR ≥200 (n = 23)	p value
	N (%)	N (%)	N (%)		N (%)	N (%)	
Pts age							
<50 yrs	21 (36.8%)	3 (5.2%)	18 (31.6%)	**<0.001**	9 (15.8%)	12 (21%)	**0.048**
>50 yrs	36 (63.2%)	22 (38.6%)	14 (24.6%)		25 (43.9%)	11 (19.3%)	
Previous taxane							
Yes	43 (75.4%)	16 (28%)	27 (47.4%)	0.076	25 (43.8%)	18 (31.6%)	0.68
No	14 (24.6%)	9 (15.8%)	5 (8.8%)		9 (15.8%)	5 (8.8%)	
Visceral disease							
Yes	37 (64.9%)	16 (28%)	21 (36.8%)	0.9	23 (40.3%)	14 (24.6%)	0.6
No	20 (35.1%)	9 (15.8%)	11 (19.4%)		11 (19.3%)	9 (15.8%)	
N. metastatic sites							
1–2	36 (63.2%)	18 (31.6%)	18 (31.6%)	0.22	23 (40.3%)	13 (22.8%)	0.39
>2	21 (36.8%)	7 (12.3%)	14 (24.5%)		11 (19.3%)	10 (17.6%)	
ChT type							
Taxane	48 (84.2%)	20 (35.1%)	28 (49.1%)	0.48*	28 (49.1%)	20 (35.1%)	0.7*
Gemcitabine	9 (15.8%)	5 (8.8%)	4 (7%)		6 (10.5%)	3 (5.3%)	
Maintenance ChT							
Yes	14 (24.6%)	10 (17.6%)	4 (7%)	**0.017**	11 (19.3%)	3 (5.3%)	0.097
No	43 (75.4%)	15 (26.3%)	28 (49.1%)		23 (40.3%)	20 (35.1%)	

Pts = patients; ChT = chemotherapy.

*Fisher’s exact test.

**Figure 1:**
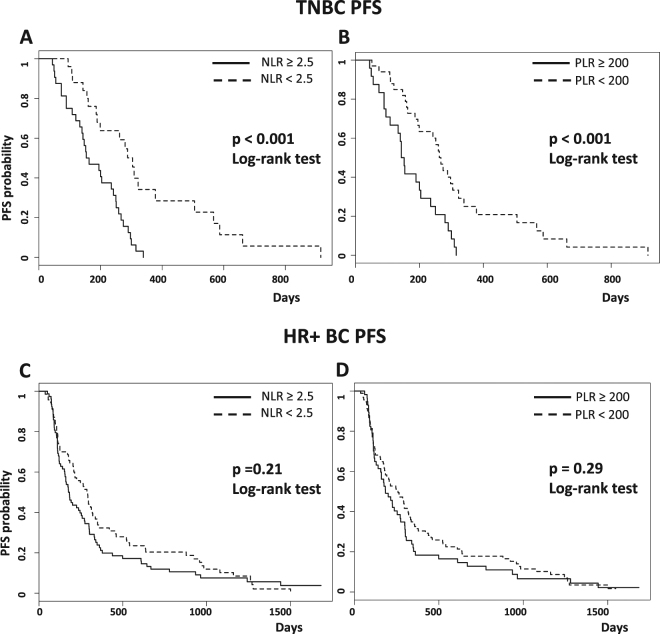
Kaplan-Meier curves of progression free survival (PFS) of TNBC and HR+ mBC patients according to baseline NLR (**A** and **C**) and PLR (**B** and **D**).

Median PFS was 304 days in patients with NLR < 2.5 and 158 days in those with NLR ≥ 2.5 (HR 3.25, 95% CI 1.72–6.25; p < 0.001) (Fig. 1A). Similar results were obtained by choosing a threshold of 3.3 (274 *vs* 148 days, p < 0.0001) or 2 (309 *vs* 186 days, p = 0.0015) (data not shown). Regarding the PLR, PFS was longer in patients with baseline PLR < 200 as compared to PLR ≥ 200 (HR 2.75, 95% CI 1.52–4.99; p < 0.001) (Fig. 1B). Baseline LMR ≥ 4.5 was also associated with significantly better PFS in TNBC patients, although the association was less strong than in the case of NLR and PLR (HR 2.22, 95% CI 1.22–4.05, p = 0.009; data not shown).

When the same parameters were evaluated before the administration of the third treatment cycle, NLR < 2.5 was still associated with reduced risk of disease progression (140 *vs* 262 days, p < 0.001), while PLR < 200 was not (259 *vs* 204 days, p = 0.48) (data not shown).

In the control population of HR+ HER2− mBC patients, mPFS was 220 days. Baseline higher NLR and PLR were non-significantly associated with the risk of disease progression (p = 0.21 and p = 0.29) (Fig. 1C and D).

### Independent predictive role of NLR and PLR on PFS

**Figure 2:**
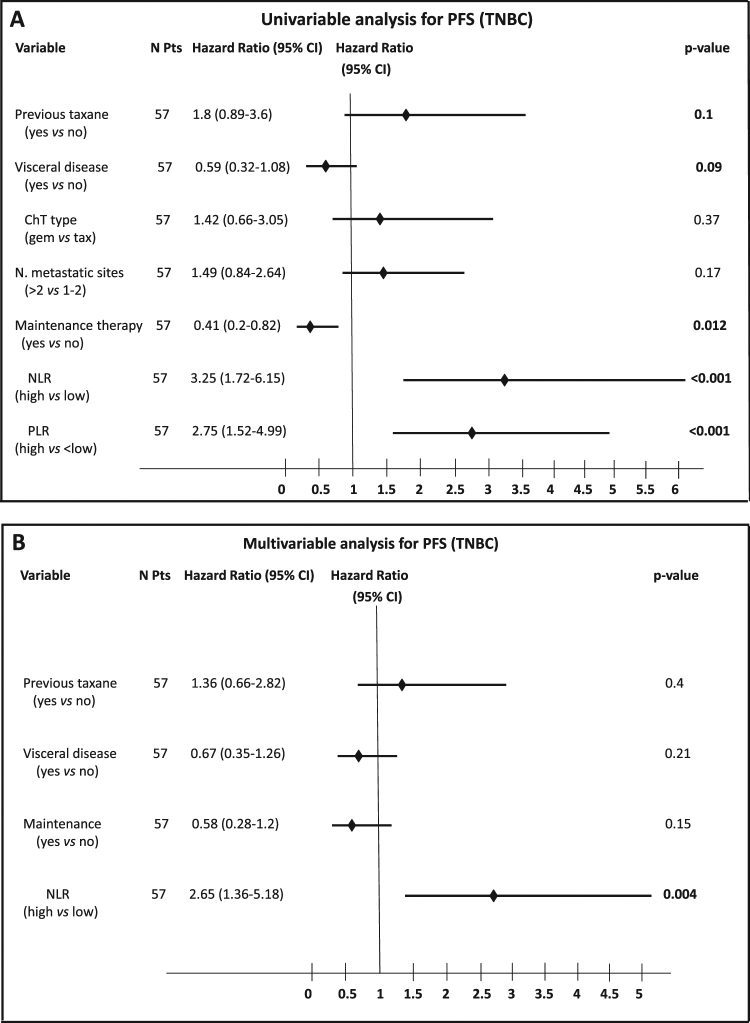
Forest plot illustrating the results of univariable (**A**) and multivariable (**B**) analysis of covariates associated with the risk of disease progression in mTNBC.

Factors associated with the risk of disease progression in mTNBC were: previous exposure to taxanes, the presence of visceral metastases, having received maintenance ChT, NLR ≥ 2.5 and PLR ≥ 200 (Fig. 2A). As previously described^27^, NLR and PLR positively correlated with each other (Pearson coefficient regression = 0.49; p < 0.001); therefore, only one of these parameters was evaluated at multivariable analysis.

In the multivariable model including NLR as a covariate, NLR ≥ 2.5 was associated with significantly lower PFS (HR 2.65; p = 0.004), while other covariates were not statistically significantly associated with PFS (Fig. 2B). When we tested PLR at multivariable analysis, PLR ≥ 200 was independently associated with poorer patient prognosis (HR 2.33; p = 0.007), while the other covariates were not, with the exception of maintenance ChT, which correlated with reduced risk of progression (HR 0.45; p = 0.027) (data not shown).

### Impact of peripheral blood parameters on OS

Median OS was 483 days in mTNBC patients and 653 days in the control population of HR+ HER2− patients.

**Figure 3:**
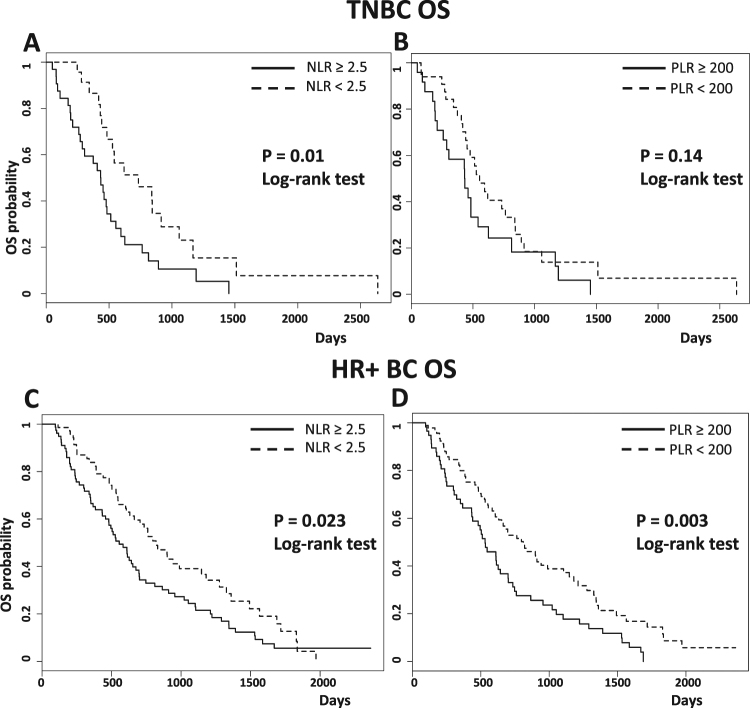
Kaplan-Meier curves of overall survival (PFS) of TNBC and HR+ mBC patients according to baseline NLR (**A** and **C**) and PLR (**B** and **D**).

In mTNBC patients, mOS was significantly longer in patients with NLR < 2.5 compared to those with NLR ≥ 2.5 (p = 0.01), while PLR values were not associated with mOS (p = 0.14) (Fig. 3, upper panels). In HR+ HER2− BC patients, both NLR < 2.5 and PLR < 200 were associated with significantly better mOS (p = 0.023 and p = 0.003, respectively) (Fig. 3, lower panels).

**Figure 4:**
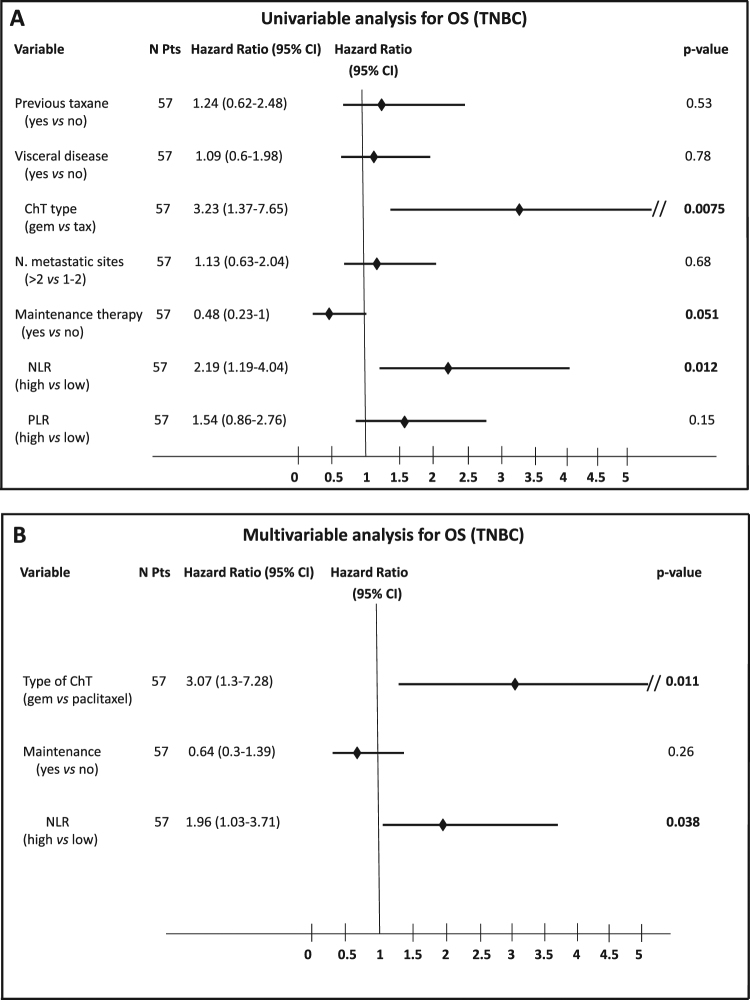
Forest plot illustrating the results of univariable (**A**) and multivariable (**B**) analysis of covariates associated with the overall survival in mTNBC.

At univariate analysis, factors associated with worse OS in mTNBC patients were NLR ≥ 2.5 and having received gemcitabine in combination with carboplatin, while maintenance ChT correlated with better PFS (Fig. 4A). At multivariable analysis, NLR ≥ 2.5 and gemcitabine treatment were independently associated with worse outcomes (Fig. 4B).

In the control population, factors associated with lower OS were: NLR ≥ 2.5, presence of visceral disease, previous taxane exposure and more advanced (>2^nd^) treatment lines, while having received maintenance therapy correlated with lower risk of death (Fig. S2, upper panel). At multivariable analysis, high NLR and visceral involvement were independently associated with lower survival, while maintenance treatment correlated with better outcome (Fig. S2, lower panel).

### Impact of germline *BRCA 1*/*2* mutations on TNBC patient PFS

Among mTNBC patients included in our study, only 19 had undergone analysis of germline mutations of the *BRCA1*/*2* genes. Of them, 6 were carriers of a pathogenic germline *BRCA1*/*2* mutation, while the remaining 13 patients had wild-type *BRCA1*/*2* gene. Median PFS was 126 days in carriers of *BRCA1*/*2* mutations and 163 days in non-mutated ones (HR = 1.09, 50% CI 0.38–3.12, p = 0.87).

## Discussion

Although platinum-based ChT is active against mTNBC, many patients fail to respond, and no biomarkers are currently available to predict treatment effectiveness. In this study, we found for the first time an association between higher NLR or PLR and worse PFS in mTNBC patients receiving carboplatin-paclitaxel or carboplatin-gemcitabine, but not in a control population of HR+ HER2− patients treated with the same regimens.

The fact that specific blood cell populations reflect the inflammatory/immune contexture is quite a well-established concept. Indeed, high neutrophils can be associated with systemic inflammation or immune suppression^30^; high platelets reflect systemic inflammation as well, but can be also associated with increased metastatization of neoplastic cells via platelet clots^30–32^; finally, low lymphocyte counts can be associated with impaired activation of adaptive immunity or poor nutritional status^33,34^. In this study, the association between clinical outcomes and NLR/PLR was stronger than in the case of individual cell counts. This is not surprising, since parameter combinations are more stable to changes in single parameters (e.g. neutrophils or platelets can increase during acute infections or glucocorticoid administration) and may capture more aspects of the tumor-immune system interplay. Notably, the HRs associated to NLR and PLR at multivariable analysis for PFS were similar, and these parameters also correlated with each other. This suggests that both NLR and PLR well reflect the inflammatory/immune contexture in mTNBC, and may be redundant as predictive biomarkers.

In TNBC patients, higher NLR correlated with lower OS, but the association was weaker than in the case of PFS; moreover, no statistically significant association was found between PLR and OS. These results can be due to the fact that, different from PFS, OS is affected by the whole treatment course, including therapies administered after platinum-based ChT; therefore, the association between NLR/PLR and PFS during platinum-based ChT could be diluted by subsequent treatments for which the same parameters are not predictive.

Conversely, both NLR and PLR correlated with lower OS in the control population of HR+ HER2− mBC patients, despite the fact that they were not predictive of PFS during platinum-based ChT. This could be due to the fact that NLR and PLR are associated to benefit from treatments administered after platinum ChT; therefore, their effect may only emerge when evaluating OS as an endpoint. Alternatively, these parameters may be generally prognostic in HR+ BC, but not predictive of benefit from specific treatments. Based on our data, we are unable to discriminate between these two hypotheses.

Studies conducted in recent years have revealed a previously unrecognized complexity of the number and functional status of different immune system subpopulations^35,36^. For instance, circulating blood lymphocytes include phenotypically and functionally different cells, such as CD8+ cytotoxic lymphocytes, regulatory T cells and exhausted lymphocytes, whose balance can determine the predominantly antitumor or protumor activity of adaptive immunity^35^. In parallel, blood monocytes include both cells that differentiate into antitumor M1 macrophages at the tissue level, and myeloid-derived suppressive cells (MDSCs) that exert pro-tumor effects by inhibiting the activity of antitumor T lymphocytes^37^. Coherently with a previous research in localized TNBC^23^, in this study we also found an association between the LMR and patient PFS, but this was weaker than in the case of NLR and PLR, and similar to the association between absolute lymphocyte counts and PFS. This finding suggests that monocytes may be poorly predictive of survival in mTNBC.

Investigating the potential association between specific immune cell populations and patient prognosis could reveal more reliable and predictive parameters to improve treatment selection in mTNBC. In this perspective, we recently started a prospective observational study to assess the role of baseline immunological parameters, as well as their on-treatment modifications, on the PFS of mTNBC patients receiving platinum-based ChT.

In this study, we did not find any significant difference in PFS duration between patients with or without *BRCA1*/*2* germline mutations, which have recently emerged as associated with higher tumor response rates during carboplatin ChT^6^. However, the number of subjects amenable to this analysis was too low, and larger studies are required to assess the independent predictive role of NLR/PLR and *BRCA1/2* mutations on the PFS of mTNBC receiving platinum-based ChT.

Strengths of this study consist in the monocentric patient cohort, which guarantees more reproducible assessment of tumor response and coherent collection of patient laboratory data by different investigators, as well as the homogeneity of ChT regimens used and the TNBC patient cohort. In particular, the fact that all TNBC patients received carboplatin-based ChT as first- or second-line treatment makes our results more robust compared to previously published data in patients treated with different regimens in different treatment lines. Weaknesses of our study consist in the retrospective design, the limited number of TNBC patients included in the analysis and the lack of data on tumor-infiltrating immune cells, which did not allow us to establish a direct, mechanistic link between peripheral blood parameters and immune cell populations in tumor microenvironment or directly infiltrating the tumor. However, the NLR and PLR calculated at the initiation of platinum-based ChT may reflect the systemic inflammatory and immunological status much more reliably than immune cells detected in tumor specimens biopsied/removed months/years before treatment administration. Moreover, the NLR and PLR could offer a more global picture of the immune contexture, thus circumventing the spatial heterogeneity in tumor-infiltrating immune cell populations.

In conclusion, the NLR and PLR are predictive of benefit from platinum-containing ChT specifically in mTNBC patients. They also confirm to have a generally prognostic role independently from tumor biology. If validated in larger prospective studies, these easy-to-measure parameters could be combined with emerging predictive biomarkers, such as germline or somatic *BRCA 1*/*2* gene mutations, to improve the selection of mTNBC patients more likely to benefit from platinum-based ChT.

## Methods

### Study setting

This was a monocentric, retrospective study on patients with mTNBC treated between July 2007 and July 2017 at Fondazione IRCCS Istituto Nazionale dei Tumori (Milan, Italy) with platinum-based ChT. The study was approved by the Institutional Review Board of Fondazione IRCCS Istituto Nazionale dei Tumori (Milano, Italy). The study was performed in accordance to relevant guidelines and regulations. Patients alive at the time of data collection and/or analysis signed an informed consent for the use of their personal data for research purposes.

Eligibility criteria were: (1) age ≥18 years; (2) pathologically or cytologically confirmed diagnosis of unresectable, locally recurrent or metastatic TNBC, as defined by ER <1% and PgR <1% expression at immunohistochemistry (IHC) analysis and an IHC score for HER2 of 0, 1+, or 2+ with negative *in situ* hybridization (ISH); (3) ECOG performance status (PS) of 0–1; (4) treatment with one the following ChT schedules: carboplatin area under the concentration-time curve (AUC) of 2 plus paclitaxel 80 mg/m^2^, or carboplatin AUC 2 plus gemcitabine 800 mg/m^2^, both given on days 1 and 8 of every-three weeks cycles; (5) availability of baseline (pre-treatment) absolute peripheral blood neutrophil, lymphocyte and platelet counts; (6) available information about previous treatment(s) for limited-stage or advanced disease; (7) available information on the date of disease progression and patient death; (8) absence of acute infections or documented bone marrow infiltration at the time of peripheral blood cell count assessment.

As a control population, we selected patients with HR+ HER2− mBC treated with the same platinum-containing regimens in the July 2007-July 2017 decade at our Institution.

All subjects fulfilling these criteria were evaluated, regardless of line of treatment for mBC.

### Objectives

The main objective of the study was to investigate the potential association between baseline NLR/PLR and clinical outcome. The primary clinical endpoint was PFS, as defined as the time between treatment initiation and disease progression or death from any cause. OS was a secondary endpoint, and was defined as the time between treatment initiation and death from any cause.

### Assessment of response

Response was assessed every 3 ChT cycles, but tumor re-evaluation was anticipated in patients with worsening symptoms or other signs suggestive of progressive disease (PD). Tumor response was evaluated according to the Response Evaluation Criteria in Solid Tumors (RECIST 1.1). Patients with only superficial, measurable disease were evaluated also clinically by measuring lesion diameters every three weeks.

### Evaluation of biomarkers

We collected data on absolute counts of peripheral blood neutrophils, lymphocytes, platelets and monocytes. We calculated the following parameters: (a) NLR by dividing neutrophil by lymphocyte counts; (b) PLR by dividing platelet by lymphocyte counts. As an exploratory analysis, we also assessed the lymphocyte-to-monocyte ratio (LMR). Blood parameters were evaluated before initiation of platinum-based ChT. Parameters measured within one month before the initiation of platinum-based ChT were considered acceptable, provided that the patient was not receiving any concomitant anticancer treatment. Patients whose blood parameters were measured more than one month before ChT initiation, or after having received the first dose of platinum-based ChT, were excluded from this study. As an exploratory analysis, we also evaluated the NLR and PLR before the administration of the third treatment cycle.

### Genetic analysis

Analysis of *BRCA1*/*2* germline mutations was carried out on the DNA extracted from peripheral blood leukocytes. All coding exons and flanking regions of BRCA1 and *BRCA2* genes were sequenced through direct sequencing, followed in most cases by multiple ligation-dependent probe amplification (MLPA) to detect large genomic rearrangements. Identified genetic variants were classified according to the IARC 5-tier scheme, following the guidelines of the Evidence-based Network for the Interpretation of Germline Mutant Alleles (ENIGMA; http://enigmaconsortium.org/)^38^.

### Statistical analysis

Patients’ characteristics were analysed by descriptive statistics. The χ^2^ or Fisher’s exact tests were used to assess the association between categorical variables, while linear correlation was used for continuous variables. PFS and OS were calculated according to the Kaplan-Meier method, and the log-rank test was used to compare survival between different patient populations. The impact of known prognostic factors on PFS was first assessed at univariate analysis. Covariates significantly associated with the risk of progression (p < 0.1) were then included in a Cox proportional hazard model to assess their independent association with survival. Based on previously published data, the following categorical covariates were tested: previous exposure to taxanes (yes *vs* no); visceral disease (yes *vs* no); the number of metastatic sites (1–2 *vs* >2); having received maintenance treatment (yes *vs* no)^9^. The type of chemotherapeutical agent combined with carboplatin (i.e. gemcitabine *vs* paclitaxel) was also tested as a covariate. All statistical analyses were performed using the software R (version 3.3.2 (2016-10-31)), while the package “survival” was used for survival analyses. A p value of 0.05 was chosen as a threshold level for statistical significance.

### Data availability

The datasets generated and/or analysed during the current study are available from the corresponding author on reasonable request.

## Electronic supplementary material

Supplementary Dataset 1
