# Supplementary material for: The neutrophil-to-lymphocyte and platelet-to-lymphocyte ratios predict efficacy of platinum-based chemotherapy in patients with metastatic triple negative breast cancer
Source: Sci Rep. 2018 Jun 7;8:8703. doi: 10.1038/s41598-018-27075-z (PMC5992181; doi:10.1038/s41598-018-27075-z)
Supplement: Supplementary file 1 — Supplementary Dataset 1 [file 41598_2018_27075_MOESM1_ESM.docx]

**The neutrophil-to-lymphocyte and platelet-to-lymphocyte ratios predict efficacy of platinum-based chemotherapy in patients with metastatic triple negative breast cancer**

Claudio Vernieri ^a,b^*, Alessia Mennitto ^a^, Michele Prisciandaro ^a^, Veronica Huber ^c^, Monica Milano ^a^, Lucia Rinaldi ^a^, Maria Silvia Cona ^a^, Claudia Maggi ^a^, Benvenuto Ferrari ^a^, Siranoush Manoukian ^d^, Gabriella Mariani ^a^, Giulia Bianchi ^a^, Giuseppe Capri ^a,^, Licia Rivoltini ^c^ and Filippo de Braud ^a,e^

^a^ *Medical Oncology Unit, Fondazione IRCCS Istituto Nazionale dei Tumori, Via Venezian 1, 20133 Milan, Italy*

^b^ *Fondazione Istituto FIRC di Oncologia Molecolare (IFOM), Via Adamello 16, Milan, Italy*

^c^ *Immunotherapy of Cancer Unit, Fondazione IRCCS Istituto Nazionale dei Tumori, Via Venezian 1, 20133 Milan, Italy*

^d^ *Medical Genetics Unit, Fondazione IRCCS Istituto Nazionale dei Tumori, Via Venezian 1, 20133 Milan, Italy*

^e^ *Universita’ degli Studi di Milano, Milan, Italy*

* *Corresponding author:* Fondazione IRCCS Istituto Nazionale dei Tumori, Via Venezian 1, 20133 Milan, Italy*. E-mail address:* [claudio.vernieri@istitutotumori.mi.it](mailto:claudio.vernieri@istitutotumori.mi.it) (C. Vernieri)
